# (*E*)-1-(2-Nitro­benzyl­idene)-2-phenyl­hydrazine

**DOI:** 10.1107/S1600536810025882

**Published:** 2010-07-07

**Authors:** Hazoor Ahmad Shad, M. Nawaz Tahir, Muhammad Ilyas Tariq, Muhammad Sarfraz, Shahbaz Ahmad

**Affiliations:** a Department of Chemistry, Bahauddin Zakariya University, Multan 60800, Pakistan; b Department of Physics, University of Sargodha, Sargodha, Pakistan; c Department of Chemistry, University of Sargodha, Sargodha, Pakistan

## Abstract

The asymmetric unit of the title compound, C_13_H_11_N_3_O_2_, contains two mol­ecules with slightly different conformations: the dihedral angle between the aromatic rings is 13.01 (10)° in one mol­ecule and 14.05 (10)° in the other. Both mol­ecules feature short intra­molecular C—H⋯O contacts, which generate *S*(6) rings. In the crystal, both mol­ecules form inversion dimers linked by pairs of N—H⋯O hydrogen bonds, thereby generating *R*_2_^2^(16) rings.

## Related literature

For background information on Schiff bases and related crystal structures, see: Mufakkar *et al.* (2010 ▶); Tahir *et al.* (2010 ▶). For graph-set notation, see: Bernstein *et al.* (1995 ▶).
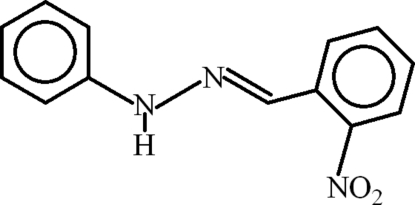


## Experimental

### 

#### Crystal data

C_13_H_11_N_3_O_2_
*M* *_r_* = 241.25
Orthorhombic, 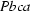

*a* = 19.4021 (13) Å
*b* = 12.1065 (7) Å
*c* = 20.0554 (11) Å
*V* = 4710.8 (5) Å^3^
*Z* = 16
Mo *K*α radiation
μ = 0.10 mm^−1^
*T* = 296 K
0.32 × 0.28 × 0.24 mm

#### Data collection

Bruker Kappa APEXII CCD diffractometer
Absorption correction: multi-scan (*SADABS*; Bruker, 2005 ▶) *T*_min_ = 0.972, *T*_max_ = 0.979
18932 measured reflections
4260 independent reflections
2577 reflections with *I* > 2σ(*I*)
*R*_int_ = 0.041

#### Refinement

*R*[*F* ^2^ > 2σ(*F* ^2^)] = 0.045
*wR*(*F* ^2^) = 0.117
*S* = 1.01
4260 reflections
325 parameters
H-atom parameters constrained
Δρ_max_ = 0.16 e Å^−3^
Δρ_min_ = −0.14 e Å^−3^

### 

Data collection: *APEX2* (Bruker, 2009 ▶); cell refinement: *SAINT* (Bruker, 2009 ▶); data reduction: *SAINT*; program(s) used to solve structure: *SHELXS97* (Sheldrick, 2008 ▶); program(s) used to refine structure: *SHELXL97* (Sheldrick, 2008 ▶); molecular graphics: *ORTEP-3 for Windows* (Farrugia, 1997 ▶) and *PLATON* (Spek, 2009 ▶); software used to prepare material for publication: *WinGX* (Farrugia, 1999 ▶) and *PLATON*.

## Supplementary Material

Crystal structure: contains datablocks global, I. DOI: 10.1107/S1600536810025882/hb5534sup1.cif

Structure factors: contains datablocks I. DOI: 10.1107/S1600536810025882/hb5534Isup2.hkl

Additional supplementary materials:  crystallographic information; 3D view; checkCIF report

## Figures and Tables

**Table 1:** Hydrogen-bond geometry (Å, °)

*D*—H⋯*A*	*D*—H	H⋯*A*	*D*⋯*A*	*D*—H⋯*A*
C7—H7⋯O1	0.93	2.24	2.773 (3)	116
C20—H20⋯O3	0.93	2.27	2.788 (3)	115
N1—H1⋯O3^i^	0.86	2.42	3.242 (2)	161
N4—H4*A*⋯O1^ii^	0.86	2.39	3.207 (2)	158

Symmetry codes: (i) 
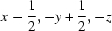
; (ii) 
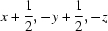
.

**Fig. 1.**
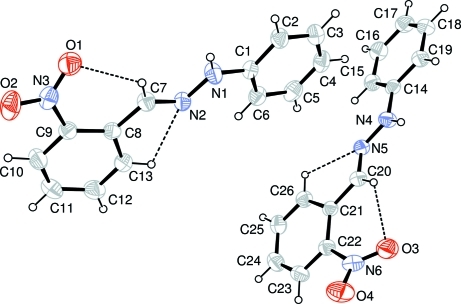
View of (I) with displacement ellipsoids drawn at the 50% probability level. H-atoms are shown by small circles of arbitrary radii.

**Fig. 2.**
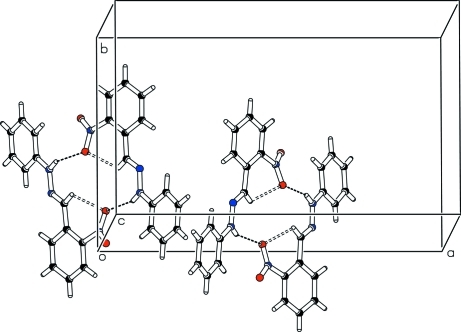
The partial packing of (I), which shows that molecules form dimers.

## supplementary crystallographic information

### Comment

We have reported crystal structures of Schiff bases containing phenylhydrazine (Mufakkar *et al.*, 2010) and 2-nitrobenzaldehyde (Tahir *et al.*, 2010) and as a part of this project, we report herein the structure and synthesis of the title compound (I, Fig. 1).

The title compound consists of two molecules in the crystallographic asymmetric unit which differ from each other geometrically. In one molecule, the phenylhydrazine A (C1—C6/N1/N2) and group B (C7—C13) of 2-nitrobenzaldehyde are planar with r. m. s deviation of 0.0228 and 0.0068 Å, respectively. The nitro group C (O1/N3/O2) is of course planar. The dihedral angle between A/B, A/C and B/C is 14.05 (10)°, 13.38 (32)° and 17.41 (30)°, respectively. In second molecule, the phenylhydrazine D (C1—C6/N1/N2) and group E (C7—C13) of 2-nitrobenzaldehyde are also planar with r. m. s deviation of 0.0054 and 0.0037 Å, respectively. The dihedral angle between D/E is 13.01 (10)°. The nitro group F (O3/N6/O4) of this molecule makes dihedral angle of 25.02 (27)° with group D, whereas it is oriented at 27.12 (27)° with group E. In each molecule there exist S(5) and S(6) ring motifs (Bernstein *et al.*, 1995) due to intramolecular H-bonding of C—H···N and C—H···O type, respectively. The molecules are stabilized in the form of dimers due to N—H···O type of H-bondings with *R*_2_^2^(16) ring motifs (Table 1, Fig. 2).

### Experimental

Equimolar quantities of phenylhydrazine and 2-nitrobenzaldehyde were refluxed in methanol for 25 min resulting in a violet solution. The solution was kept at room temperature, which afforded violet prisms of (I) after 48 h.

### Refinement

The H-atoms were positioned geometrically (N–H = 0.86, C–H = 0.93 Å) and refined as riding with *U*_iso_(H) = *xU*_eq_(C, N), where *x* = *x* = 1.2 for all H-atoms.

### Crystal data

**Table:**

C_13_H_11_N_3_O_2_	*F*(000) = 2016
*M**_r_* = 241.25	*D*_x_ = 1.361 Mg m^−^^3^
Orthorhombic, *P**b**c**a*	Mo *K*α radiation, λ = 0.71073 Å
Hall symbol: -P 2ac 2ab	Cell parameters from 2577 reflections
*a* = 19.4021 (13) Å	θ = 2.2–25.3°
*b* = 12.1065 (7) Å	µ = 0.10 mm^−^^1^
*c* = 20.0554 (11) Å	*T* = 296 K
*V* = 4710.8 (5) Å^3^	Prism, violet
*Z* = 16	0.32 × 0.28 × 0.24 mm

### Data collection

**Table:**

Bruker Kappa APEXII CCD diffractometer	4260 independent reflections
Radiation source: fine-focus sealed tube	2577 reflections with *I* > 2σ(*I*)
graphite	*R*_int_ = 0.041
Detector resolution: 8.20 pixels mm^-1^	θ_max_ = 25.3°, θ_min_ = 2.2°
ω scans	*h* = −21→23
Absorption correction: multi-scan (*SADABS*; Bruker, 2005)	*k* = −13→14
*T*_min_ = 0.972, *T*_max_ = 0.979	*l* = −24→24
18932 measured reflections	

### Refinement

**Table:**

Refinement on *F*^2^	Primary atom site location: structure-invariant direct methods
Least-squares matrix: full	Secondary atom site location: difference Fourier map
*R*[*F*^2^ > 2σ(*F*^2^)] = 0.045	Hydrogen site location: inferred from neighbouring sites
*wR*(*F*^2^) = 0.117	H-atom parameters constrained
*S* = 1.01	*w* = 1/[σ^2^(*F*_o_^2^) + (0.0426*P*)^2^ + 0.9743*P*] where *P* = (*F*_o_^2^ + 2*F*_c_^2^)/3
4260 reflections	(Δ/σ)_max_ < 0.001
325 parameters	Δρ_max_ = 0.16 e Å^−^^3^
0 restraints	Δρ_min_ = −0.14 e Å^−^^3^

### Special details

**Table:**

Geometry. Bond distances, angles *etc*. have been calculated using the rounded fractional coordinates. All su's are estimated from the variances of the (full) variance-covariance matrix. The cell e.s.d.'s are taken into account in the estimation of distances, angles and torsion angles
Refinement. Refinement of *F*^2^ against ALL reflections. The weighted *R*-factor *wR* and goodness of fit *S* are based on *F*^2^, conventional *R*-factors *R* are based on *F*, with *F* set to zero for negative *F*^2^. The threshold expression of *F*^2^ > σ(*F*^2^) is used only for calculating *R*-factors(gt) *etc*. and is not relevant to the choice of reflections for refinement. *R*-factors based on *F*^2^ are statistically about twice as large as those based on *F*, and *R*- factors based on ALL data will be even larger.

### Fractional atomic coordinates and isotropic or equivalent isotropic displacement parameters (Å^2^)

**Table:**

	*x*	*y*	*z*	*U*_iso_*/*U*_eq_	
O1	−0.02767 (9)	0.48801 (15)	−0.11736 (8)	0.0811 (7)	
O2	−0.03824 (10)	0.64989 (16)	−0.15815 (9)	0.0982 (8)	
N1	0.12471 (9)	0.27574 (14)	−0.00241 (8)	0.0646 (7)	
N2	0.12334 (8)	0.38691 (13)	0.00220 (8)	0.0546 (6)	
N3	−0.01209 (9)	0.58595 (18)	−0.11891 (9)	0.0642 (7)	
C1	0.15768 (10)	0.21311 (16)	0.04626 (10)	0.0519 (7)	
C2	0.15290 (12)	0.09962 (17)	0.04246 (10)	0.0634 (8)	
C3	0.18603 (12)	0.03400 (19)	0.08869 (12)	0.0709 (9)	
C4	0.22378 (12)	0.0800 (2)	0.13901 (11)	0.0709 (10)	
C5	0.22787 (12)	0.1929 (2)	0.14304 (11)	0.0716 (10)	
C6	0.19503 (11)	0.26019 (18)	0.09740 (10)	0.0621 (8)	
C7	0.08784 (11)	0.44017 (16)	−0.04126 (10)	0.0568 (8)	
C8	0.08420 (10)	0.56020 (16)	−0.03663 (9)	0.0505 (7)	
C9	0.03909 (11)	0.62879 (17)	−0.07170 (9)	0.0528 (7)	
C10	0.03841 (13)	0.74245 (19)	−0.06341 (12)	0.0724 (9)	
C11	0.08391 (15)	0.79155 (19)	−0.02040 (13)	0.0799 (10)	
C12	0.12983 (13)	0.7266 (2)	0.01410 (12)	0.0764 (10)	
C13	0.13014 (11)	0.61467 (18)	0.00641 (10)	0.0621 (8)	
O3	0.53109 (9)	0.28838 (15)	0.13060 (9)	0.0901 (8)	
O4	0.52867 (11)	0.44681 (16)	0.08338 (9)	0.1054 (8)	
N4	0.37407 (9)	0.07450 (14)	0.24028 (8)	0.0580 (6)	
N5	0.38069 (8)	0.18402 (13)	0.25032 (8)	0.0529 (6)	
N6	0.51143 (10)	0.38409 (18)	0.12819 (9)	0.0680 (8)	
C14	0.33940 (10)	0.00973 (16)	0.28709 (10)	0.0501 (7)	
C15	0.30960 (11)	0.05488 (18)	0.34361 (10)	0.0608 (8)	
C16	0.27540 (12)	−0.0131 (2)	0.38795 (11)	0.0713 (9)	
C17	0.27064 (12)	−0.1249 (2)	0.37713 (12)	0.0723 (9)	
C18	0.30009 (12)	−0.16936 (18)	0.32096 (12)	0.0706 (9)	
C19	0.33432 (11)	−0.10272 (17)	0.27593 (11)	0.0610 (8)	
C20	0.41511 (10)	0.23954 (16)	0.20722 (10)	0.0539 (7)	
C21	0.42235 (10)	0.35828 (16)	0.21663 (9)	0.0487 (7)	
C22	0.46637 (10)	0.42733 (17)	0.18061 (10)	0.0527 (7)	
C23	0.47060 (12)	0.53979 (18)	0.19184 (11)	0.0642 (8)	
C24	0.43038 (13)	0.58790 (19)	0.23970 (12)	0.0719 (9)	
C25	0.38560 (12)	0.5228 (2)	0.27580 (11)	0.0678 (9)	
C26	0.38167 (11)	0.41148 (18)	0.26445 (10)	0.0596 (8)	
H1	0.10513	0.24344	−0.03557	0.0775*	
H2	0.12723	0.06716	0.00856	0.0761*	
H3	0.18261	−0.04244	0.08555	0.0851*	
H4	0.24630	0.03560	0.16995	0.0851*	
H5	0.25332	0.22489	0.17727	0.0858*	
H6	0.19807	0.33661	0.10112	0.0746*	
H7	0.06484	0.40278	−0.07511	0.0682*	
H10	0.00707	0.78539	−0.08706	0.0868*	
H11	0.08374	0.86777	−0.01461	0.0959*	
H12	0.16112	0.75952	0.04309	0.0917*	
H13	0.16181	0.57300	0.03047	0.0745*	
H4A	0.39117	0.04455	0.20509	0.0696*	
H15	0.31260	0.13038	0.35166	0.0729*	
H16	0.25524	0.01739	0.42579	0.0856*	
H17	0.24775	−0.16993	0.40748	0.0868*	
H18	0.29697	−0.24492	0.31318	0.0848*	
H19	0.35401	−0.13356	0.23798	0.0732*	
H20	0.43505	0.20467	0.17068	0.0647*	
H23	0.50081	0.58273	0.16684	0.0770*	
H24	0.43318	0.66341	0.24777	0.0862*	
H25	0.35776	0.55483	0.30822	0.0814*	
H26	0.35083	0.36969	0.28943	0.0716*	

### Atomic displacement parameters (Å^2^)

**Table:**

	*U*^11^	*U*^22^	*U*^33^	*U*^12^	*U*^13^	*U*^23^
O1	0.0805 (12)	0.0809 (12)	0.0818 (12)	−0.0026 (10)	−0.0189 (9)	−0.0115 (9)
O2	0.0928 (14)	0.1218 (15)	0.0801 (12)	0.0250 (12)	−0.0258 (10)	0.0245 (11)
N1	0.0801 (14)	0.0509 (11)	0.0628 (11)	0.0090 (9)	−0.0199 (10)	−0.0048 (8)
N2	0.0537 (11)	0.0525 (11)	0.0575 (10)	0.0061 (8)	−0.0015 (9)	−0.0006 (8)
N3	0.0593 (12)	0.0829 (14)	0.0504 (11)	0.0128 (11)	0.0009 (9)	0.0032 (10)
C1	0.0478 (12)	0.0550 (13)	0.0530 (12)	0.0077 (10)	−0.0007 (10)	0.0004 (10)
C2	0.0649 (15)	0.0613 (14)	0.0640 (14)	0.0058 (12)	−0.0117 (11)	−0.0015 (11)
C3	0.0731 (16)	0.0616 (14)	0.0781 (16)	0.0093 (12)	−0.0048 (14)	0.0072 (12)
C4	0.0629 (16)	0.0837 (19)	0.0660 (15)	0.0187 (13)	−0.0016 (12)	0.0156 (12)
C5	0.0583 (15)	0.097 (2)	0.0595 (14)	0.0071 (13)	−0.0102 (11)	−0.0014 (13)
C6	0.0562 (14)	0.0670 (14)	0.0632 (14)	0.0044 (11)	−0.0053 (11)	−0.0060 (11)
C7	0.0614 (14)	0.0557 (14)	0.0533 (12)	0.0019 (11)	−0.0049 (11)	−0.0015 (10)
C8	0.0495 (13)	0.0528 (13)	0.0492 (11)	−0.0008 (10)	0.0046 (10)	0.0046 (9)
C9	0.0532 (13)	0.0572 (13)	0.0479 (12)	−0.0021 (10)	0.0013 (10)	0.0052 (9)
C10	0.0797 (18)	0.0590 (15)	0.0784 (16)	0.0070 (13)	0.0002 (14)	0.0158 (12)
C11	0.098 (2)	0.0505 (14)	0.0912 (18)	−0.0078 (14)	0.0007 (16)	0.0030 (13)
C12	0.0789 (18)	0.0650 (16)	0.0854 (17)	−0.0130 (14)	−0.0085 (14)	−0.0043 (13)
C13	0.0575 (14)	0.0613 (14)	0.0674 (14)	−0.0010 (11)	−0.0072 (12)	0.0035 (11)
O3	0.0841 (13)	0.0784 (12)	0.1079 (14)	−0.0043 (10)	0.0345 (10)	−0.0129 (10)
O4	0.1200 (16)	0.1156 (15)	0.0807 (12)	−0.0243 (12)	0.0354 (11)	0.0206 (11)
N4	0.0609 (12)	0.0537 (11)	0.0593 (10)	−0.0076 (9)	0.0082 (9)	0.0003 (8)
N5	0.0471 (10)	0.0521 (11)	0.0596 (10)	−0.0030 (8)	−0.0027 (8)	0.0050 (8)
N6	0.0617 (13)	0.0785 (14)	0.0639 (12)	−0.0201 (11)	0.0049 (10)	0.0003 (11)
C14	0.0411 (11)	0.0551 (13)	0.0540 (12)	−0.0017 (10)	−0.0054 (10)	0.0088 (10)
C15	0.0600 (14)	0.0611 (14)	0.0612 (13)	0.0017 (11)	−0.0018 (11)	0.0040 (11)
C16	0.0699 (16)	0.0852 (18)	0.0588 (14)	0.0048 (14)	0.0074 (12)	0.0083 (13)
C17	0.0656 (16)	0.0809 (18)	0.0705 (15)	−0.0069 (13)	0.0057 (13)	0.0224 (13)
C18	0.0674 (16)	0.0575 (14)	0.0870 (17)	−0.0081 (12)	0.0041 (14)	0.0106 (12)
C19	0.0576 (14)	0.0597 (14)	0.0657 (13)	−0.0019 (11)	0.0077 (11)	0.0022 (10)
C20	0.0495 (13)	0.0569 (13)	0.0554 (12)	−0.0045 (10)	0.0039 (10)	0.0017 (10)
C21	0.0440 (12)	0.0529 (12)	0.0493 (11)	−0.0018 (10)	−0.0045 (10)	0.0055 (9)
C22	0.0500 (12)	0.0585 (13)	0.0497 (12)	−0.0034 (10)	−0.0035 (10)	0.0027 (10)
C23	0.0676 (15)	0.0598 (15)	0.0653 (14)	−0.0136 (12)	−0.0092 (12)	0.0119 (11)
C24	0.0839 (18)	0.0525 (14)	0.0793 (16)	0.0009 (13)	−0.0142 (14)	−0.0009 (12)
C25	0.0714 (16)	0.0646 (16)	0.0675 (14)	0.0105 (12)	0.0007 (13)	−0.0037 (12)
C26	0.0556 (14)	0.0627 (15)	0.0606 (13)	0.0006 (11)	0.0033 (11)	0.0041 (10)

### Geometric parameters (Å, °)

**Table:**

O1—N3	1.224 (3)	C5—H5	0.9300
O2—N3	1.215 (3)	C6—H6	0.9300
O3—N6	1.221 (3)	C7—H7	0.9300
O4—N6	1.223 (3)	C10—H10	0.9300
N1—C1	1.392 (3)	C11—H11	0.9300
N1—N2	1.349 (2)	C12—H12	0.9300
N2—C7	1.285 (3)	C13—H13	0.9300
N3—C9	1.467 (3)	C14—C19	1.383 (3)
N1—H1	0.8600	C14—C15	1.385 (3)
N4—N5	1.347 (2)	C15—C16	1.382 (3)
N4—C14	1.396 (3)	C16—C17	1.374 (3)
N5—C20	1.283 (3)	C17—C18	1.373 (3)
N6—C22	1.464 (3)	C18—C19	1.381 (3)
N4—H4A	0.8600	C20—C21	1.457 (3)
C1—C2	1.379 (3)	C21—C26	1.399 (3)
C1—C6	1.379 (3)	C21—C22	1.397 (3)
C2—C3	1.380 (3)	C22—C23	1.382 (3)
C3—C4	1.366 (3)	C23—C24	1.367 (3)
C4—C5	1.372 (3)	C24—C25	1.379 (3)
C5—C6	1.381 (3)	C25—C26	1.369 (3)
C7—C8	1.458 (3)	C15—H15	0.9300
C8—C9	1.397 (3)	C16—H16	0.9300
C8—C13	1.405 (3)	C17—H17	0.9300
C9—C10	1.386 (3)	C18—H18	0.9300
C10—C11	1.370 (4)	C19—H19	0.9300
C11—C12	1.375 (4)	C20—H20	0.9300
C12—C13	1.364 (3)	C23—H23	0.9300
C2—H2	0.9300	C24—H24	0.9300
C3—H3	0.9300	C25—H25	0.9300
C4—H4	0.9300	C26—H26	0.9300
			
N2—N1—C1	120.29 (16)	C11—C10—H10	120.00
N1—N2—C7	117.70 (16)	C10—C11—H11	120.00
O1—N3—O2	122.04 (19)	C12—C11—H11	120.00
O1—N3—C9	119.56 (18)	C13—C12—H12	120.00
O2—N3—C9	118.4 (2)	C11—C12—H12	119.00
N2—N1—H1	120.00	C12—C13—H13	119.00
C1—N1—H1	120.00	C8—C13—H13	119.00
N5—N4—C14	119.88 (16)	N4—C14—C15	122.00 (18)
N4—N5—C20	117.69 (16)	N4—C14—C19	118.58 (18)
O4—N6—C22	118.0 (2)	C15—C14—C19	119.42 (19)
O3—N6—C22	119.83 (19)	C14—C15—C16	119.5 (2)
O3—N6—O4	122.2 (2)	C15—C16—C17	121.2 (2)
C14—N4—H4A	120.00	C16—C17—C18	119.2 (2)
N5—N4—H4A	120.00	C17—C18—C19	120.5 (2)
N1—C1—C6	122.54 (18)	C14—C19—C18	120.2 (2)
N1—C1—C2	118.24 (18)	N5—C20—C21	118.69 (18)
C2—C1—C6	119.22 (19)	C22—C21—C26	115.09 (18)
C1—C2—C3	120.3 (2)	C20—C21—C22	125.64 (18)
C2—C3—C4	120.8 (2)	C20—C21—C26	119.26 (18)
C3—C4—C5	118.8 (2)	C21—C22—C23	122.79 (19)
C4—C5—C6	121.5 (2)	N6—C22—C21	121.51 (18)
C1—C6—C5	119.4 (2)	N6—C22—C23	115.70 (19)
N2—C7—C8	118.89 (18)	C22—C23—C24	120.0 (2)
C7—C8—C9	126.23 (18)	C23—C24—C25	119.0 (2)
C9—C8—C13	115.33 (18)	C24—C25—C26	120.7 (2)
C7—C8—C13	118.44 (18)	C21—C26—C25	122.4 (2)
C8—C9—C10	122.40 (19)	C14—C15—H15	120.00
N3—C9—C10	114.96 (19)	C16—C15—H15	120.00
N3—C9—C8	122.63 (18)	C15—C16—H16	119.00
C9—C10—C11	120.0 (2)	C17—C16—H16	119.00
C10—C11—C12	119.1 (2)	C16—C17—H17	120.00
C11—C12—C13	120.9 (2)	C18—C17—H17	120.00
C8—C13—C12	122.2 (2)	C17—C18—H18	120.00
C3—C2—H2	120.00	C19—C18—H18	120.00
C1—C2—H2	120.00	C14—C19—H19	120.00
C4—C3—H3	120.00	C18—C19—H19	120.00
C2—C3—H3	120.00	N5—C20—H20	121.00
C3—C4—H4	121.00	C21—C20—H20	121.00
C5—C4—H4	121.00	C22—C23—H23	120.00
C6—C5—H5	119.00	C24—C23—H23	120.00
C4—C5—H5	119.00	C23—C24—H24	121.00
C5—C6—H6	120.00	C25—C24—H24	120.00
C1—C6—H6	120.00	C24—C25—H25	120.00
C8—C7—H7	121.00	C26—C25—H25	120.00
N2—C7—H7	121.00	C21—C26—H26	119.00
C9—C10—H10	120.00	C25—C26—H26	119.00
			
C1—N1—N2—C7	−174.85 (18)	C7—C8—C13—C12	−179.5 (2)
N2—N1—C1—C2	174.72 (18)	C9—C8—C13—C12	1.0 (3)
N2—N1—C1—C6	−5.8 (3)	C13—C8—C9—C10	−1.6 (3)
N1—N2—C7—C8	178.67 (17)	N3—C9—C10—C11	179.9 (2)
O1—N3—C9—C8	17.2 (3)	C8—C9—C10—C11	1.2 (3)
O1—N3—C9—C10	−161.6 (2)	C9—C10—C11—C12	0.0 (4)
O2—N3—C9—C8	−163.5 (2)	C10—C11—C12—C13	−0.6 (4)
O2—N3—C9—C10	17.8 (3)	C11—C12—C13—C8	0.0 (4)
N5—N4—C14—C15	1.6 (3)	N4—C14—C15—C16	179.44 (19)
N5—N4—C14—C19	−179.04 (18)	C19—C14—C15—C16	0.1 (3)
C14—N4—N5—C20	177.50 (18)	N4—C14—C19—C18	−179.7 (2)
N4—N5—C20—C21	179.35 (17)	C15—C14—C19—C18	−0.3 (3)
O3—N6—C22—C23	152.6 (2)	C14—C15—C16—C17	0.3 (3)
O4—N6—C22—C21	153.5 (2)	C15—C16—C17—C18	−0.4 (4)
O3—N6—C22—C21	−27.2 (3)	C16—C17—C18—C19	0.2 (4)
O4—N6—C22—C23	−26.7 (3)	C17—C18—C19—C14	0.1 (3)
C6—C1—C2—C3	−1.0 (3)	N5—C20—C21—C22	169.55 (19)
N1—C1—C6—C5	−178.38 (19)	N5—C20—C21—C26	−11.8 (3)
N1—C1—C2—C3	178.5 (2)	C20—C21—C22—N6	−0.5 (3)
C2—C1—C6—C5	1.1 (3)	C20—C21—C22—C23	179.7 (2)
C1—C2—C3—C4	0.3 (3)	C26—C21—C22—N6	−179.12 (18)
C2—C3—C4—C5	0.4 (3)	C26—C21—C22—C23	1.1 (3)
C3—C4—C5—C6	−0.3 (3)	C20—C21—C26—C25	−179.8 (2)
C4—C5—C6—C1	−0.5 (3)	C22—C21—C26—C25	−1.0 (3)
N2—C7—C8—C9	−167.98 (19)	N6—C22—C23—C24	179.8 (2)
N2—C7—C8—C13	12.6 (3)	C21—C22—C23—C24	−0.3 (3)
C7—C8—C9—N3	0.3 (3)	C22—C23—C24—C25	−0.5 (3)
C7—C8—C9—C10	178.9 (2)	C23—C24—C25—C26	0.5 (4)
C13—C8—C9—N3	179.78 (18)	C24—C25—C26—C21	0.3 (3)

### Hydrogen-bond geometry (Å, °)

**Table:**

*D*—H···*A*	*D*—H	H···*A*	*D*···*A*	*D*—H···*A*
C7—H7···O1	0.93	2.24	2.773 (3)	116
C20—H20···O3	0.93	2.27	2.788 (3)	115
N1—H1···O3^i^	0.86	2.42	3.242 (2)	161
N4—H4A···O1^ii^	0.86	2.39	3.207 (2)	158

Symmetry codes: (i) *x*−1/2, −*y*+1/2, −*z*; (ii) *x*+1/2, −*y*+1/2, −*z*.
